# *MUNC18–1* gene abnormalities are involved in neurodevelopmental disorders through defective cortical architecture during brain development

**DOI:** 10.1186/s40478-017-0498-5

**Published:** 2017-11-30

**Authors:** Nanako Hamada, Ikuko Iwamoto, Hidenori Tabata, Koh-ichi Nagata

**Affiliations:** 1 Department of Molecular Neurobiology, Institute for Developmental Research, Aichi Human Service Center, 713-8 Kamiya, Kasugai, Aichi 480-0392 Japan; 2 Research Fellow of Japan Society for the Promotion of Science, Tokyo, Japan; 3 Department of Neurochemistry, Nagoya University Graduate School of Medicine, Nagoya, Japan

**Keywords:** Munc18–1, Syntaxin1A, Corticogenesis, Neurodevelopmental disorders

## Abstract

While Munc18–1 interacts with Syntaxin1 and controls the formation of soluble N-ethylmaleimide-sensitive factor attachment protein receptors (SNARE) complex to regulate presynaptic vesicle fusion in developed neurons, this molecule is likely to be involved in brain development since its gene abnormalities cause early infantile epileptic encephalopathy with suppression-burst (Ohtahara syndrome), neonatal epileptic encephalopathy and other neurodevelopmental disorders. We thus analyzed physiological significance of Munc18–1 during cortical development. Munc18–1-knockdown impaired cortical neuron positioning during mouse corticogenesis. Time-lapse imaging revealed that the mispositioning was attributable to defects in radial migration in the intermediate zone and cortical plate. Notably, Syntaxin1A was critical for radial migration downstream of Munc18–1. As for the underlying mechanism, Munc18–1-knockdown in cortical neurons hampered post-Golgi vesicle trafficking and subsequent vesicle fusion at the plasma membrane in vivo and in vitro, respectively. Notably, Syntaxin1A-silencing did not affect the post-Golgi vesicle trafficking. Taken together, Munc18–1 was suggested to regulate radial migration by modulating not only vesicle fusion at the plasma membrane to distribute various proteins on the cell surface for interaction with radial fibers, but also preceding vesicle transport from Golgi to the plasma membrane. Although knockdown experiments suggested that Syntaxin1A does not participate in the vesicle trafficking, it was supposed to regulate subsequent vesicle fusion under the control of Munc18–1. These observations may shed light on the mechanism governing radial migration of cortical neurons. Disruption of Munc18–1 function may result in the abnormal corticogenesis, leading to neurodevelopmental disorders with *MUNC18–1* gene abnormalities.

**Electronic supplementary material:**

The online version of this article (10.1186/s40478-017-0498-5) contains supplementary material, which is available to authorized users.

## Introduction

Munc18 (mammalian homologs of *Caenorhabditis elegans* uncoordinated-18) proteins are essential regulators of exocytosis, and mammals express 3 highly homologous isoforms, Munc18–1~3. While Munc18–1 is primarily expressed in neurons and neuroendocrine cells [1, 2], Munc-18-2 and Munc-18-3 are expressed widely including brain [3–5]. As for the function, Munc18–1 regulates neuronal exocytosis by serving as a molecular chaperone for Syntaxin1, and controls SNARE (soluble *N*-ethylmaleimide-sensitive factor attachment protein receptor) complex formation through modulating Syntaxin1 level at the plasma membrane [6]. On the other hand, Munc18–3, which has been intensively analyzed for the regulation of insulin-mediated GLUT4 (glucose transporter) localization in adipocytes, seems to be involved in corticogenesis [7].

Based on the reports of gene abnormalities including haploinsufficiency and heterozygous mutations, an essential role of MUNC-18-1 has been predicted in the etiology of early infantile epileptic encephalopathy with suppression-burst (EIEE; Ohtahara syndrome) and other neurodevelopmental disorders such as neonatal epileptic encephalopathy (NEE), intellectual disability (ID) and autism spectrum disorders (ASD) [8–16]. Although molecular mechanism underlying the etiology of disorders with *MUNC-18-1* (also known as *STXBP1*) gene abnormalities remains to be elucidated, the abovementioned reports may indicate that MUNC18–1 regulates excitatory neuron positioning and synapse network formation during brain development to establish the cortical architecture. In this context, abnormal positioning of cortical neurons (a focal cortical dysplasia type 1a) has been observed in an autopsy sample from an epilepsy patient with a *MUNC18–1* mutation (c.1631G > T; p.Gly544Val) [10].

While intensive studies have been performed for the role of Munc18–1 in the neurotransmitter release of differentiated neurons, little is known about its physiological role during corticogenesis. Pathophysiological significance of *MUNC18–1* mutations in neurodevelopmental disorders also remains to be enigmatic, although EIEE-causative mutations may render MUNC-18-1 thermolabile with a strong propensity to aggregate [12]. These mutants are assumed to form aggregates with wild type MUNC-18-1, leading to proteasomal degradation, and thus lower the level of functional MUNC-18-1 [17, 18].

Newborn cortical neurons generated in the VZ are primarily multipolar, and exhibit slow and irregular movement in the lower IZ for ~24 h. Then, they transform into a bipolar shape with a leading process and an axon in the IZ, and perform radial migration toward the pial surface [19, 20]. Strict spatiotemporal regulation of neuron migration is crucial for brain development and intellectual functioning. In this study, we found that Munc-18-1 as well as Syntaxin1A is indispensable for radial migration of cortical neuron during brain development. Munc18–1 was shown to regulate post-Golgi transport of vesicles to the plasma membrane as well as subsequent vesicle fusion at cell surface for proper distribution of proteins crucial for neuron migration, while Syntaxin1A appeared to be involved in the vesicle fusion as a downstream effector of Munc18–1. Thus, differential roles of Munc18–1 and Syntaxin1A may be critical for cortical neuron migration. Protein kinase C(PKC)-mediated phosphorylation seemed to be required for the migration regulation. Then, epilepsy-related *MUNC18–1* gene abnormalities were suggested to induce a loss-of-function which may cause defective neuronal migration, leading to abnormal cytoarchitecture of cerebral cortex associated with the clinical manifestations of patients with the gene abnormalities.

## Materials and methods

### Study approval

We followed the Fundamental Guidelines for Proper Conduct of Animal Experiments and Related Activity in Academic Research Institution under the jurisdiction of the Ministry of Education, Culture, Sports, Science and Technology, Japan. All of the protocols for animal handling and treatment were reviewed and approved by the Animal Care and Use Committee of Institute for Developmental Research, Aichi Human Service Center (Approval number, M10).

### Plasmid construction

Mouse (m) Munc18–1 clone (*mStxbp1*) was kindly provided from Dr. T. Izumi (Gunma Univ., Japan) [21]. pCAG-HA-N-Cadherin was from Dr. T. Kawauchi (Inst. Biomed. Res. Innov., Kobe, japan). mMunc18–2, mMunc18–3, mSyntaxin1A and mSyntaxin1B were amplified by RT-PCR with adult mouse brain RNA pool. These cDNAs were constructed into pCAG-Myc vector (Addgene Inc., Cambridge, MA). The following target sequences were inserted into pSuper-puro RNAi vector (OligoEngine, Seattle, WA); mMunc18–1#1, GGACATTGGCACAAGAATA (1558–1574), mMunc18–1#2, GAGGATGAACACTGGCGAG (942–960) and mStx1A#1, GGACATTGGCACAAGAATA (mSyntaxin1A, 1558–1574). Numbers indicate the positions from translational start sites. It should be noted that we could prepare only one RNAi vector specific for mSyntaxin1A, since Syntaxin1A and 1B are small proteins (288 aa) with high homology (84%). For RNAi-resistant versions of mMunc18–1 and mSyntaxin1A, mMunc18–1R and mSyntaxin1A, respectively, silent mutations were introduced in the target sequences as underlined (ATGGGCACTGGCATAAAAACA in mMunc18–1#1; ATGGGCACTGGCATAAAAACA in mStx1A#1). For the control RNAi experiments, we used pSuper-H1.shLuc designed against luciferase (CGTACGCGGAATACTTCGA) [22]. By the use of pCAG-GFP-mMunc18–1R as a template, site-directed mutagenesis was performed with KOD-Plus Mutagenesis kit (Toyobo, Osaka, Japan) to generate Munc18–1 mutants at PKC- and Cyclin-dependent kinase 5(Cdk5)-phosphorylation sites (Ser306/Ser313 and Thr574, respectively), causative mutants for EIEE (c.539G > A, p.C180Y; c.1217G > A**,** p.R406H; c.1328 T > G, p.M443R) and NEE (c.1631G > T; p.G544 V). Chimeric cDNA, mSyntaxin1AB, containing N- and C-terminal regions of mSyntaxin1A (aa1–149) and B (aa145–255), respectively, was constructed by PCR. All constructs were verified by DNA sequencing.

### Antibodies

Mouse monoclonal anti-Munc18–1, anti-Syntaxin1A, anti-nestin and anti-N-Cadherin antibodies were purchased from Abnova Inc. (H00006812-M01; Taipei, Taiwan), Synaptic Systems (110,111; Gottingen, Germany), R&D Systems (MAB2736; Minneapolis, MN) and BD Biosciences (610,920; San Jose, CA), respectively. Mouse monoclonal anti-Myc (#192–3) and polyclonal rabbit anti-GFP (#598) were from MBL Inc. (Nagoya, Japan). Polyclonal rabbit anti-Myc and anti-Sept11 were prepared as previously described [23, 24]. Rabbit polyclonal anti-RFP, anti-HA (hemagglutinin) and anti-N-Cadherin recognizing the extracellular domain were from Rockland Immunochemicals Inc. (#600–401-379; Gilbertsville, PA) and Santa Cruze Biotech. (sc-805 and sc-7939; Santa Cruze, CA), respectively. Polyclonal anti-Tag-1 and monoclonal anti-GM130 were from R&D systems (AF4439; Minneapolis, MN) and BD Biosciences (610,822; San Jose, CA), respectively.

### Cell culture, transfection and immunofluorescence

COS7 cells were cultured essentially as previously described [25] and transfected with Lipofectamine 2000 **(**Life Technologies Japan, Tokyo**)** according to the manufacturer’s instruction. Immunofluorescence analyses were done as previously described [26]. Alexa Fluor 488- or 568-labeled IgG **(**Life Technologies Japan**)** was used as a secondary antibody. 4′,6-diamidino-2-phenylindole (DAPI) was used for DNA staining. Fluorescent images were obtained using FV-1000 confocal laser microscope (Olympus, Tokyo, Japan).

### In utero electroporation

In utero electroporation was performed with pregnant ICR mice essentially as previously described [27]. Briefly, expression plasmids and/or pSuper-RNAi plasmid were injected with pCAG-GFP or pCAG-RFP (red fluorescent protein) into the lateral ventricles of embryos, followed by electroporation using CUY21 electroporator (NEPA Gene, Chiba, Japan) with 50 ms of 35 V electronic pulse for 5 times with 450 ms intervals. As for quantitative analyses of neuronal migration, distribution of GFP- or RFP-positive cells was quantified by calculation of the number of labeled cells in each region of the brain slices [22, 28].

### Time-lapse imaging

After in utero electroporation, organotypic coronal slices (250 μm thickness) were prepared with a microtome from the anterior third of the forebrain at indicated time points, placed on insert membranes, mounted in collagen gel as previously described [29, 30]. The dishes were then cultured in a CO_2_ incubator chamber (5% CO_2_, at 37 °C) fitted onto the confocal laser microscope, and the dorsomedial region of the neocortex was examined. Approximately 8–15 optical Z sections were acquired every 5 to 15 min for 24 h, and about 10 focal planes (50-μm thickness) were merged to visualize the entire shape of the cells.

### EdU (5-ethynil-2′-deoxyuridine) incorporation experiments

pCAG-GFP vector was electroporated in utero into embryos with control or shMunc#1 at embryonic day (E)14. Forty h after electroporation, pregnant mice were given an intraperitoneal injection of EdU at 25 mg/kg body weight. One h after injection, brains were fixed with 4% paraformaldehyde and frozen sections were made. GFP and EdU were detected with anti-GFP and Alexa Fluor555 azide (Life Technologies Japan), respectively.

### Statistical analysis

Results were expressed as means ± SD. When data were obtained from only two groups, Student’s and Welch’s t test were used for comparison. For other experiments, the rate of cell scores was initially analyzed using the one-way analysis of variance (ANOVA). Subsequently, Tukey-Kramer least significant difference (LSD) test was applied to absolute values as a post hoc test of multiple comparisons. The level of statistical significance was considered to be *p* < 0.05. Statistical analysis was performed using Statcel3 software (OMS Inc., Tokorozawa, Japan).

## Results

### Distribution of Munc18–1 in embryonic mouse brain

**Fig. 1:**
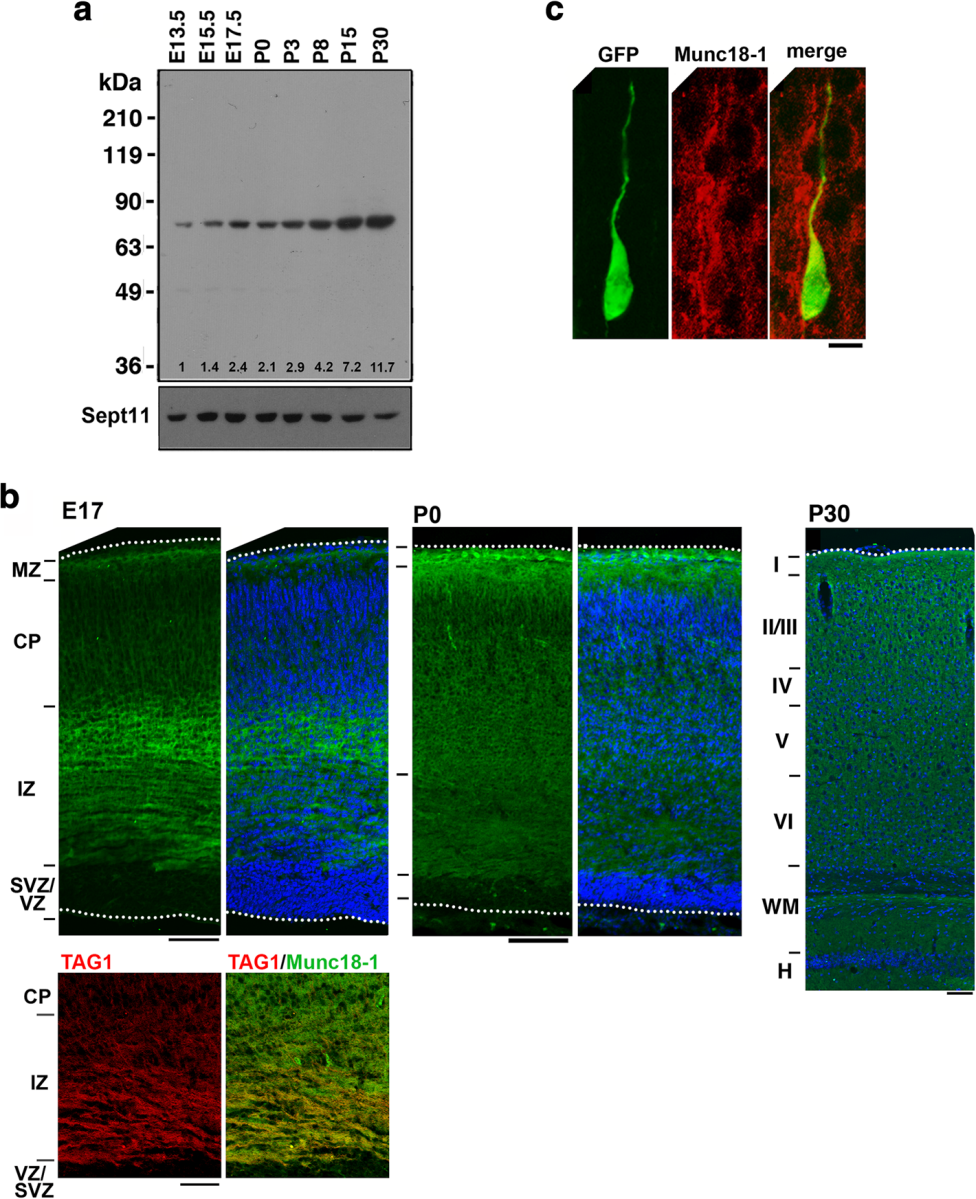
Expression of Munc18–1 in developing mouse brain. **a** Developmental changes of Munc18–1 protein amounts. Whole lysates (20 μg protein) of cerebral cortices at various developmental stages were subjected to western blotting (10% gel) with anti-Munc18–1. Anti-Sept11 was used for a loading control. The expression level of Munc18–1 was corrected based on that of Sept11 using ImageJ software, and relative expression was shown as fold-increase over the expression level at E13.5. **b** Munc18–1 distribution in developing cerebral cortex. Coronal sections were examined for Munc18–1 (green) and nuclei (blue) by immunohistochemical staining at E17, P0 and P30. Bars, 100 μm. A cortical slice (E17) was double-stained for Munc18–1 (green) and Tag-1 (red). Tag-1 distribution and a merged image were shown. Bar, 50 μm. **c** Subcellular distribution of Munc18–1 in migrating neurons in the CP. pCAG-GFP was electroporated into cerebral cortices at E14.5 and fixed at E17 to visualize migrating neurons. Coronal sections were prepared and stained for GFP and Munc18–1. A representative neuron in the lower CP was displayed. Bar, 5 μm

Involvement of MUNC18–1 in the etiology of neurodevelopmental disorders implicates its physiological role in brain development. When Munc18–1 expression during mouse corticogenesis was examined by western blotting, it was detected from E13.5 and gradually increased throughout the developmental process analyzed until postnatal day (P)30 (Fig 1a). The expression profile was correlated with that of the northern blotting [31]. In immunohistochemical analyses, Munc18–1 was detected mainly in the intermediate zone (IZ) where axons are enriched and glia cells including oligodendrocytes are not yet present at E17 and P0 (Fig. 1b, Additional file 1: Figure S1). On the other hand, Munc18–1 was detected moderately in the cortical plate (CP) during corticogenesis while it was barely detected in the progenitor and stem cells in the ventricular zone (VZ)/subventricular zone (SVZ) throughout the development (Fig 1b). Notably, Munc18–1 was distributed uniformly in the cerebral cortex in the adult brain (P30) (Fig 1b). These results were consistent with those of in situ hybridization, where Munc18–1 was expressed in CP neurons [32]. Further analyses revealed that Munc18–1 was distributed in the cytosol of bipolar cells committed to layer II-III pyramidal neurons (Fig 1c), which were still migrating in the lower part of CP at E17 as described previously [33]. This result suggests that Munc18–1 participates in radial migration during corticogenesis.

### Roles of Munc18–1 in excitatory neuron migration during corticogenesis

**Fig. 2:**
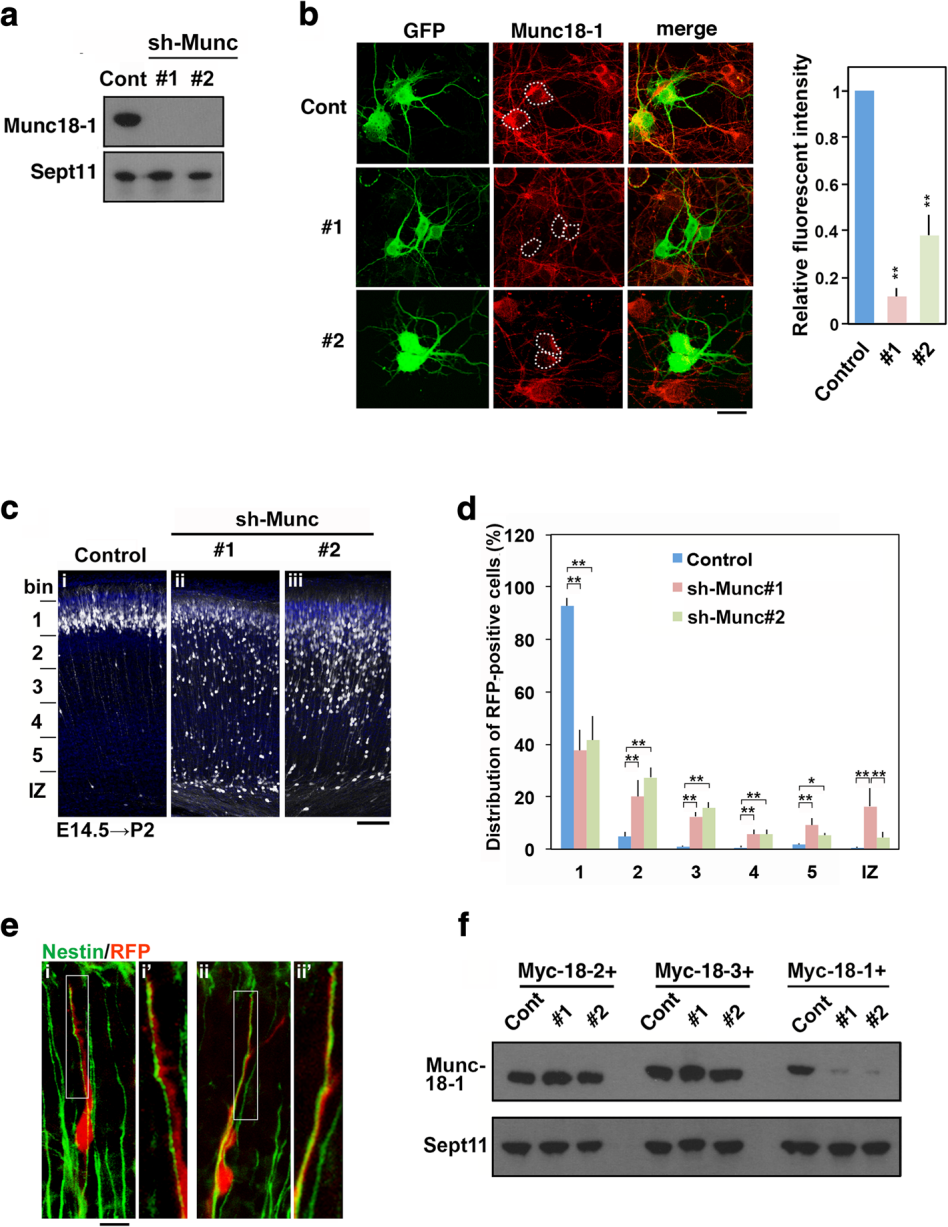
Role of Munc18–1 in neuronal migration during mouse brain development. **a** Characterization of shMunc vectors. pCAG-Myc-mMunc18–1 was transfected into COS7 cells with pSuper-H1.shLuc (Cont), sh-Munc#1 or #2. After 48 h, cells were harvested and subjected to western blotting (10% gel) with anti-Myc (Munc18–1). Anti-Sept11 was used for a loading control. **b** Knockdown of endogenous Munc18–1 in cortical neurons. pCAG-GFP was transfected with pSuper-H1.shLuc (Cont), shMunc#1 or #2 into dissociated neurons obtained at E14 and cultured for 48 h. Then, cells were fixed and immunostained for GFP (green) and Munc18–1 (red). Merged images were also shown. Bar, 10 μm. The fluorescent signals of Munc18–1 in the cell body enclosed with dotted lines were measured by ImageJ software. The ratio of Munc18–1 signal of knockdown cell to that of control one was calculated (*n* = 30 cells each). **p < 0.01 by Student’s *t*-test. **c** Migration defects of Munc18–1-deficient cortical neurons. pCAG-RFP was electroporated in utero with pSuper-H1.shLuc (Cont), sh-Munc#1 or #2 into E14.5 embryonic brains. Coronal sections were prepared at P2 and stained with anti-RFP (white) and DAPI (blue). Bar, 100 μm. **d** Quantification of the distribution of Munc18–1-deficient neurons in distinct parts of the cortex (bin 1–5, and IZ) for each condition shown in (**c**). Error bars indicate SD (Control, *n* = 5; shMunc18–1#1, *n* = 8; shMunc18–1#2, *n* = 4); **p < 0.01 *p < 0.05 by Tukey-Kramer LSD. **e** Morphology of Munc18–1-deficient migrating neurons at E18. After transfection with shMunc#1 with pCAG-RFP at E14.5, coronal sections were stained for nestin (green) and RFP (red). Images of the indicated areas in **i** and **ii** were shown at higher magnification in **i’** and **ii’**, respectively. Bar, 5 μm. **f** Effects of sh-Munc#1 and #2 on Munc18–2 and Munc18–3 expression. pCAG-Myc-mMunc18–1, −mMunc18–2 or -mMunc18–3 was transfected into COS7 cells with pSuper-H1.shLuc (Cont), sh-Munc#1 or #2. Analyses were done as in (**a**)

Since Munc18–1 is likely to be involved in the lamination of cerebral cortex during brain development (Fig 1), we examined the role of Munc18–1 in migration of newly generated cortical neurons. We constructed 2 RNAi vectors, pSuper-mMunc18–1(sh-Munc)#1 and #2, which efficiently knocked down Munc18–1 overexpressed in COS7 cells (Fig 2a). These vectors also silenced endogenous Munc18–1 in primary cultured cortical neurons (Fig 2b). Then, pCAG-RFP was electroporated in utero with pSuper-H1.shLuc (Control), sh-Munc#1 or #2 into progenitor and stem cells in the VZ of E14.5 mice brains. When localization of transfected cells and their progeny was visualized at P2, RFP-positive neurons were positioned normally at the superficial layer (bin 1; layers II ~ III) of CP in the control slice (Fig. 2c–d). In sharp contrast, a considerable portion of cells transfected with the RNAi vectors remained in the lower zone of CP and IZ (Fig. 2c,ii,iii and d). Meanwhile, many Munc18–1-deficient neurons still reached the superficial layer of CP (Fig. 2c, ii, iii and d), perhaps due to incomplete depletion of Munc18–1 in neurons incorporating low amount of the RNAi vector; knockdown effects in each cell may vary according to the cell surface area physically exposed to the ventricular lumen from which RNAi vectors pass into cells. Since cell shape is closely associated with cell migration, we examined the morphology of Munc18–1-deficient neurons and found it indistinguishable from normal cells. The deficient neurons apparently formed a normal leading process and attached to radial glial fibers (Fig 2e). We could not examine long-term effects of Munc18–1-knockdown (sh-Munc#1 and #2) since the deficient neurons disappeared at P7 (data not shown), presumably due to cell death as in the case of hippocampal neurons from Munc18–1-null mice [34]. Consistently, caspase3 activation was detected in Munc18–1-deficient neurons at P3 but not during radial migration (E17.5) (data not shown). Notably, brain magnetic resonance imaging (MRI) revealed cortical atrophy in patients with *MUNC18–1* mutations, although it is not clear if this atrophy is attributable to the neuronal cell death [35].

Since Munc18–3 is also involved in corticogenesis [7], we asked if the observed migration defects are indeed ascribed to Munc18–1-silencing. Consequently, neither sh-Munc#1 nor #2 silenced mMunc18–3 as well as mMunc18–2 in COS7 cells, strongly suggesting that the observed migration defects were caused by Munc18–1 knockdown (Fig 2f).

At the end of radial migration, the migratory mode changes to the terminal translocation, a crucial step for the completion of neuronal migration, just beneath the marginal zone (MZ) [36]. When we asked if Munc18–1-knockdown affects the terminal translocation, it was completed under the conditions where Munc18–1 was silenced (Additional file 2: Figure S2). The deficient neurons could enter the outermost region of the CP termed primitive cortical zone, and the tip of the leading process was attached to the MZ. These results indicate that Munc18–1 is not involved in the terminal translocation.

**Fig. 3:**
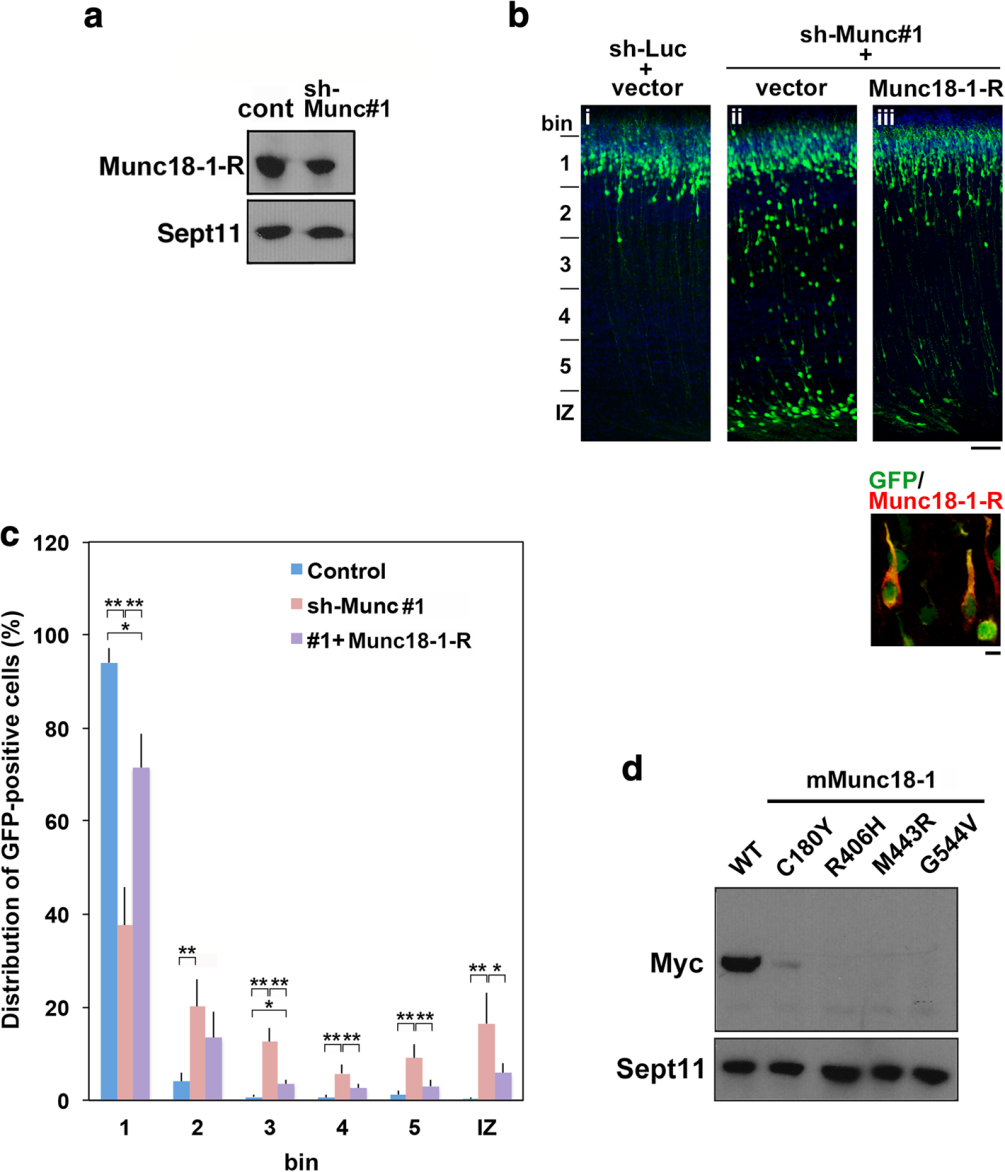
Rescue of Munc18–1-knockdown-induced migration defects. **a** Characterization of an RNAi-resistant mMunc18–1, mMunc18–1R. pCAG-Myc-mMunc18–1R was cotransfected into COS7 cells with pSuper-H1.shLuc (Cont) or shMunc#1. Western blotting analyses were done as Fig. 2a. **b** pCAG-GFP was electroporated with pSuper-H1.shLuc + pCAG-Myc **(i)**, with sh-Munc#1 + pCAG-Myc **(ii)** or sh-Munc#1 + pCAG-Myc-mMunc18–1R **(iii)** into cerebral cortices at E14.5, followed by fixation at P2. Coronal sections were stained for GFP (green) and nuclei (blue) **(i-iii)**. Bar, 100 μm. Myc-tagged Munc18–1-R expression in **(iii)** was confirmed (*lower* panel). Bar, 5 μm. **c** Quantification of the distribution of neurons in distinct regions for each condition in (**b**). Error bars indicate SD (**i**, n = 5; **ii**, n = 4; **iii**, *n* = 7); ** *p* < 0.01; * *p* < 0.05, by Tukey-Kramer LSD. **d** Expression of Myc-Munc18–1 (WT) and Myc-tagged mutants (C180Y, R406H, M443R, G544 V) in COS7 cells. After 48 h of transfection, cells were harvested and subjected to western blotting (10% gel) with anti-Myc. Anti-Sept11 was used for a loading control

Rescue experiments were performed to rule out off-target effects. To this end, Munc18–1R was prepared that was resistant to sh-Munc#1-mediated silencing in COS7 cells (Fig 3a). When pCAG-GFP and shMunc#1 were electroporated with pCAG-Myc-mMunc18–1R, the positional defects were rescued at P2 (Fig. 3b and c), indicating that the mispositioning observed was indeed caused by Munc18–1-knockdown. On the other hand, effects of expression of epilepsy-causative mutants (mMunc18–1-C180Y, −R406H, −M443R or -G544 V) could not be determined since these mutants were presumed to be degradated in cortical neurons as in the case of Neuro2A, PC12 and COS cells (Fig. 3d) [12, 18], suggestive of pathophysiological significance of *MUNC18–1* haploinsufficiency for the abnormal cortical neuron migration.

### Time-lapse imaging of migration of Munc18–1-deficient neurons in cortical slices

**Fig. 4:**
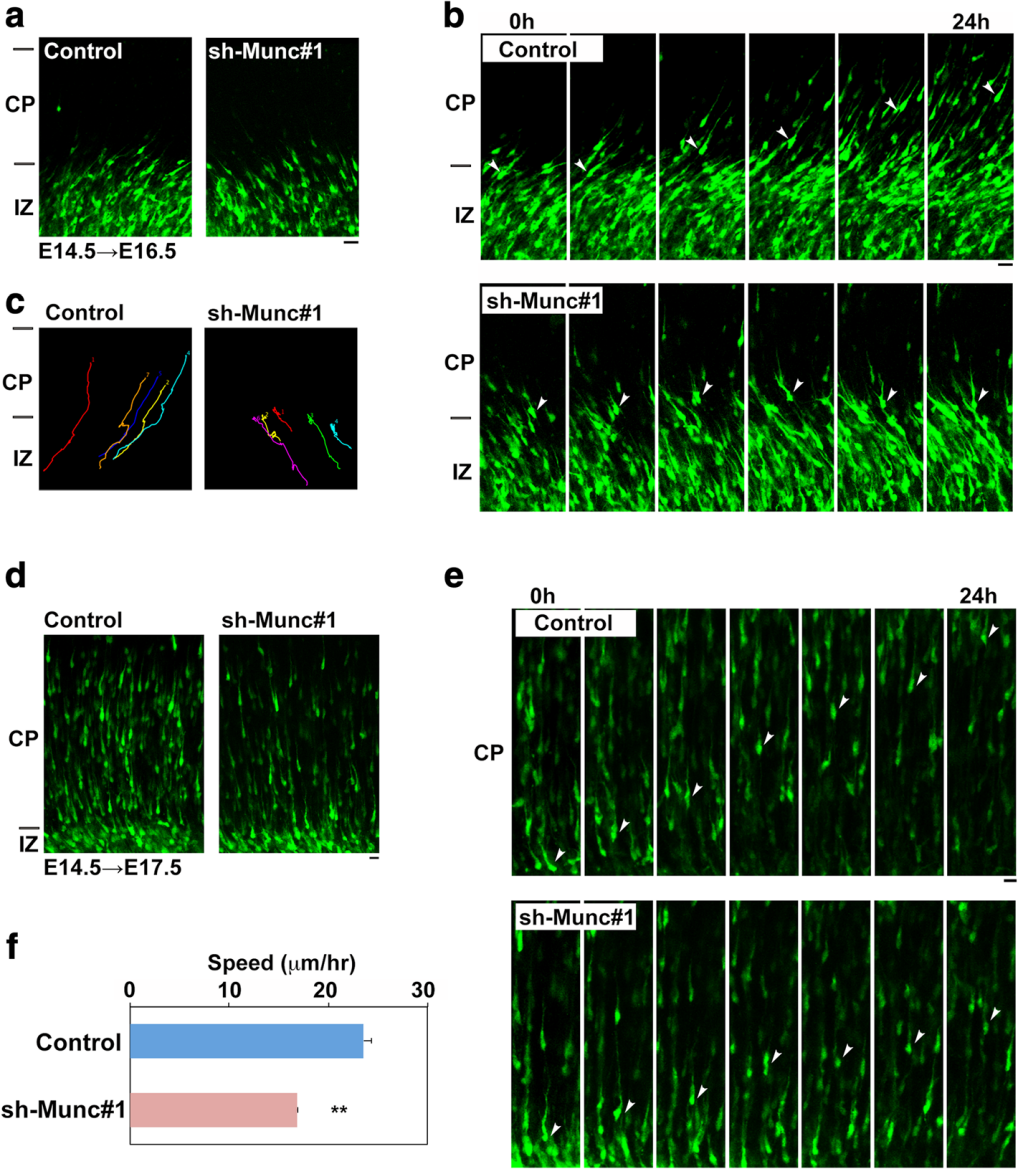
Time-lapse imaging of Munc18–1-deficient neuron migration. Experiments were repeated three times for each case, and the migration pattern was observed with the confocal microscope for 10 cells in each imaging. Representative results were shown in (**a**-**e**). **a** Cortical slices at the beginning of tissue culture. E14.5 cortices were electroporated with pCAG-GFP together with pSuper-H1.shLuc (Control) or sh-Munc#1, followed by coronal section slice preparation at E16.5 and time-lapse imaging. There was no difference in transfection efficiency between the experiments. Bars in (**a, b, d**) and (**e**), 20 μm. **b** Time-lapse imaging of control and Munc18–1-deficient (sh-Munc#1) neurons stranded around IZ in electroporated cortical slices. **c** Tracing of control or Munc18–1-deficient neurons in upper IZ - lower CP in (**b**). Migratory tracks of 5 cells were traced, and demonstrated as color lines with numbering. **d** Cortical slices at the beginning of time-lapse imaging (E17.5) of neurons migrating in the CP. **e** Time-lapse imaging of control and Munc18–1-deficient neurons migrating in the CP. **f** Migration speed of control and the deficient (sh-Munc#1) neurons in the middle-upper CP. Seventeen - 27 cells were analyzed in each experiment (n = 3). Error bars indicate SD; **p < 0.01 by Student’s *t*-test

We carried out time-lapse imaging of Munc18–1-deficient cortical neurons migrating in the IZ and CP. VZ cells were electroporated with pCAG-GFP together with control RNAi vector or sh-Munc#1 at E14.5. At the beginning of imaging (E16.5), Munc18–1-deficient neurons displayed multipolar shape similar to the control cells and some cells were transforming into bipolar neurons (Fig. 4a), whereas abnormal phenotypes of the deficient cells came to be observed when time-lapse imaging was continued.

In the control experiment, GFP-positive neurons normally transformed from multipolar to bipolar in the upper IZ and moved into the CP. Then, typical radial migration was visualized toward the pial surface (Fig. 4b *upper* panels, C *left* panel and Additional file 3: Video 1). On the contrary, Munc18–1-deficient cells frequently remained stranded in the upper IZ - lower CP during the imaging time period (~24 h) and the migration was significantly prevented during the analyses (Fig. 4b, *lower* panels, c *right* panel and Additional file 4: Video 2). Although the multipolar-bipolar shape transition was normal, the deficient cells could not pass the IZ-CP boundary efficiently.

On the other hand, considerable population of Munc18–1-deficient cells crossed the IZ-CP border. They, however, frequently showed an abnormal migration phenotype in the CP. Although the deficient cells were morphologically indistinguishable from control cells and maintained normal bipolar polarity from the beginning of the imaging (E17.5) (Fig. 4d and e), they displayed a migration delay with a reduced speed after entering the CP (Fig. 4e, *lower* panels, f and Additional files 5 and 6: Videos 3 and 4).

As for the aberrant migration phenotype, it was notably less characteristic than knockdown phenotypes of other genes. For example, SIL1 (a chaperone-related protein)-deficient neurons exhibited abnormal bipolar-multipolar shape turnover during radial migration, while Rbfox1(an RNA-splicing factor)-deficient neurons showed a stepwise migration profile [22, 37]. Meanwhile, Nr1d1 (a circadian clock protein)-knockdown neurons displayed characteristic phenotypes such as jump in a tangential direction and reverse-directed migration [38].

Collectively, Munc18–1 was supposed to be involved in two steps of excitatory neuron migration during corticogenesis; crossing of the IZ-CP boundary and radial migration in the CP. While migration defects may occur at the IZ-CP boundary when the RNAi effect is strong, delayed locomotion in the CP may occur when the RNAi effect is relatively weak.

### Munc18–1 does not regulate neuronal progenitor proliferation

Since prolonged cell cycle results in a migration delay of newly generated neurons [39], we looked into the effect of Munc18–1-silencing on the cell cycle of progenitor and stem cells in the VZ/SVZ. When S-phase cells were labeled with EdU to detect DNA synthesis, Munc18–1-deficient cells entered S-phase to a similar extent as the control cells (Additional file 7: Figure S3). These results indicate that the cell cycle G1-progression rate was not statistically different between the control and the deficient cells, and that Munc18–1-silencing did not affect cell proliferation in the VZ/SVZ. Also, positioning of EdU/GFP-double positive cells in the VZ/SVZ was not affected by the knockdown (Additional file 7: Figure S3a). Taken together with the data that Munc18–1 was barely detected in the VZ/SVZ (Fig 1b), we conclude that the neuronal positioning defects by Munc18–1-knockdown were most likely to be caused by aberrant migration.

### Effects of PKC- and Cdk5-mediated phosphorylation of Munc18–1 on cortical neuron migration

Cdk5 is known to play a central role in neuronal migration during corticogenesis [40] and phosphorylate Munc18–1 at Thr574 to regulate neuronal secretion through the modulation of interaction with Syntaxin 1A [41, 42]. Meanwhile, PKC-mediated phosphorylation of Munc18–1 at Ser306 and Ser313 has been reported to be crucial for its localization in nerve terminals [43], interaction with Syntaxin1A [44] and neurotransmitter release [45]. Analyses with chemical inhibitors suggest possible involvement of PKC in radial migration [46]. We thus analyzed the role(s) of PKC as well as Cdk5 in Munc18–1-mediated cortical neuron migration by examining whether Munc18–1 mutants at the phosphorylation sites by these kinases rescue the knockdown phenotype. As for Cdk5, both Munc18–1-T574D and -T574A mimicking phosphorylated and unphosphorylated states, respectively, rescued the migration defects (Additional file 8: Figure S4a). In contrast, no mutations at the PKC sites mimicking phosphorylated and unphosphorylated states could rescue the abnormal migration phenotype (Additional file 8: Figure S4b). Expression of each mutant was confirmed immunohistochemically (Additional file 8: Figure S4c).

### Important role of Munc18–1-Syntaxin1A interaction in cortical neuron migration

**Fig. 5:**
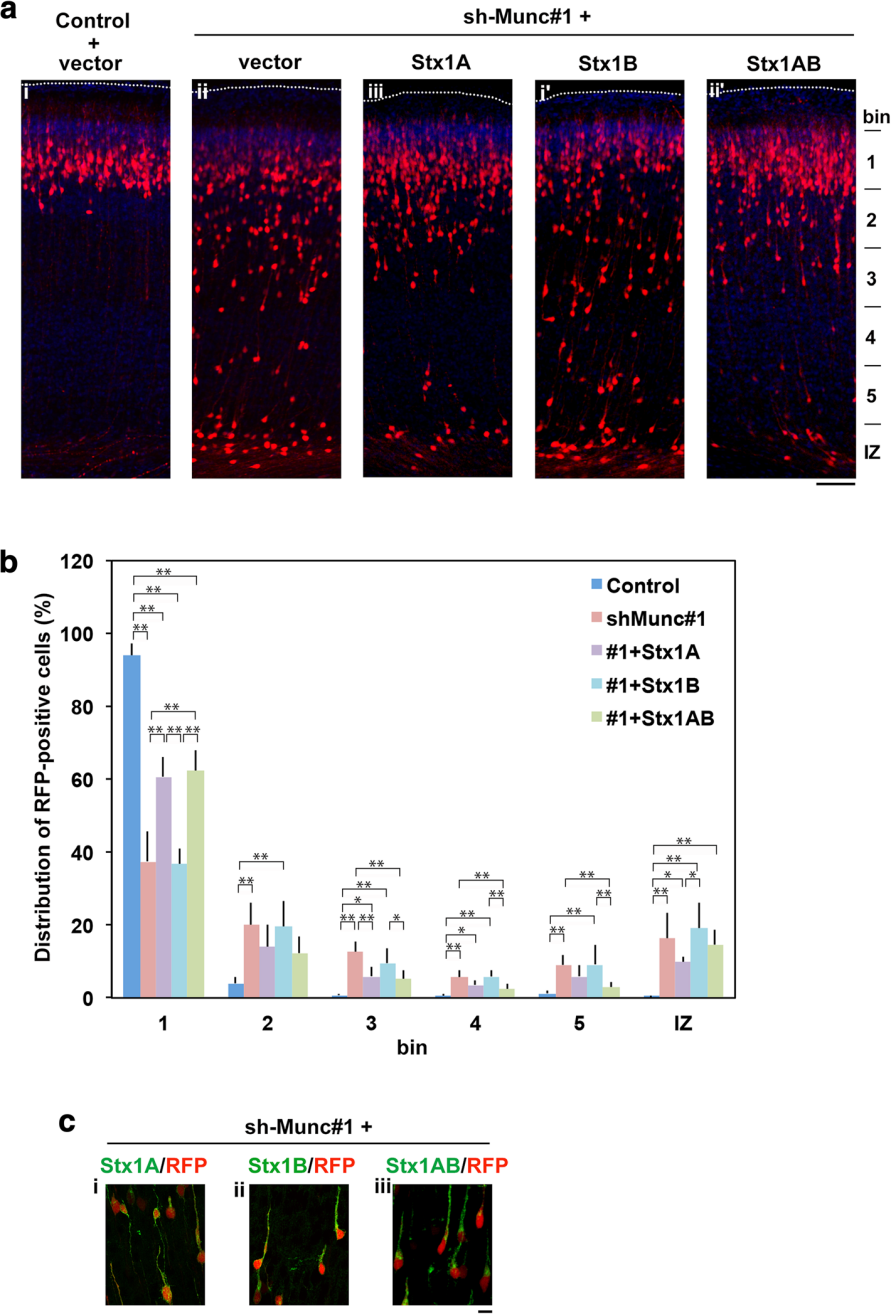
Rescue of Munc18–1-knockdown-induced migration defects by Syntaxin1A**. a** pCAG-RFP was coelectroporated in utero with pSuper-H1.shLuc (Control) + pCAG-Myc vector **(i)**, or with sh-Munc#1 together with pCAG-Myc **(ii)**, −Myc-Syntaxin1A **(iii)**, −Myc-Syntaxin1B **(i’)** or -Myc-Syntaxin1AB chimera **(ii’)** into VZ cells at E14.5, followed by fixation at P2. Coronal sections were stained for RFP (red) and nuclei (blue). Dotted lines represent the pial surface. Bar, 100 μm. **b** Quantification of the distribution of RFP-positive neurons in distinct regions of the cerebral cortex for each condition in (**a**). Error bars indicate SD (**i**, n = 5; **ii**, n = 7; **iii**, n = 7; **i’**, n = 5; **ii’**, *n* = 10); *p < 0.05, **p < 0.01 by Tukey-Kramer LSD. **c** Detection of Myc-Syntaxin1A **(i)**, −Syntaxin1B **(ii)** and -Syntaxin1AB chimera **(iii)**. Electroporation was done as in (**a**). After fixation, cells were immunostained for RFP (red) and Myc (green). Bar, 10 μm

Since Munc18–1 is a regulator for Syntaxin1 in the neurotransmitter release pathway, Syntaxin1 is also possible to regulate neuronal migration downstream of Munc18–1. Interestingly, we found that Syntaxin1A but not 1B rescued the migration defects by Munc18–1-knockdown (Fig. 5a and b), although the rescue effect was less than that by Munc18–1R (Fig. 3b and c). Coordinated function of Munc18–1 with Syntaxin1A was thus suggested to be essential for neuronal migration during corticogenesis. Notably, interaction of Munc18–1 with N-terminal region of Syntaxin1A (aa1–149) might be critical since Syntaxin1AB chimera rescued the migration defects (Fig. 5a–b). Expression of Myc-Syntaxin1A, 1B and AB chimera was confirmed (Fig 5c).

### Regulation of cortical neuron migration by Syntaxin1A

**Fig. 6:**
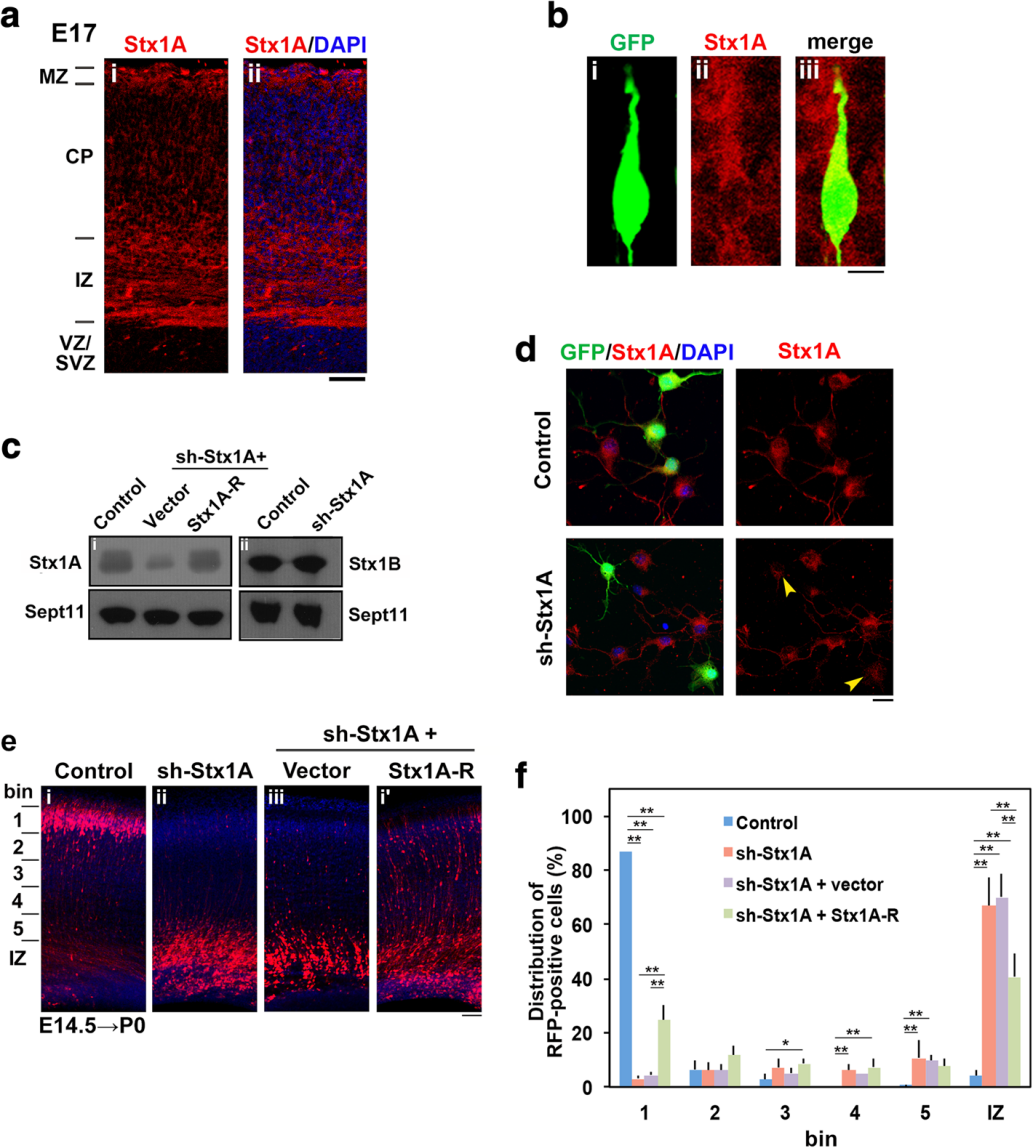
Role of Syntaxin1A in neuronal migration during corticogenesis**. a** Syntaxin1A distribution at E17. Coronal section was double-stained for Syntaxin1A **(i** and **ii)** and nuclei **(ii)**. Bar, 100 μm. **b** Subcellular distribution of Syntaxin1A in migrating neurons in the CP. pCAG-GFP was electroporated into cerebral cortices at E14.5 and fixed at E17. A coronal section was prepared and stained for GFP **(i)** and Syntaxin1A **(ii)**. Merged image was also shown **(iii)**. Bar, 5 μm. **c** Characterization of vectors. pCAG-Myc-mSyntaxin1A was transfected into COS7 cells with pSuper-H1.shLuc (Control), sh-Stx1A + pCAG-Myc (vector), or sh-Stx1A + pCAG-Myc-Syntaxin1A-R **(i)**. pCAG-Myc-mSyntaxin1B was transfected with pSuper-H1.shLuc (Control) or sh-Stx1A **(ii)**. After 48 h, cells were harvested and subjected to western blotting (10% gel) with anti-Myc. Anti-Sept11 was used for a loading control. **d** Knockdown of endogenous Syntaxin1A in cortical neurons. pCAG-GFP was transfected with pSuper-H1.shLuc (Control), or sh-Stx1A into dissociated neurons obtained at E14 and cultured for 48 h. Then, cells were fixed and immunostained for GFP (green) and Syntaxin1A (red). Nuclei were visualized by DAPI. Bar, 10 μm. **e** Migration defects of Syntaxin1A-deficient cortical neurons and rescue experiments. pCAG-RFP was electroporated in utero with pSuper-H1.shLuc **(i)**, sh-Stx1A **(ii)**, sh-Stx1A + pCAG-Myc **(iii)** or sh-Stx1A + pCAG-Myc-Syntaxin1A-R **(i’)** into E14.5 embryonic brains. Coronal sections were prepared at P0 and stained with anti-RFP (red) and DAPI (blue). Bar, 100 μm. **f** Quantification of the distribution of neurons in bin 1–5 and IZ in (**e**). Error bars indicate SD (**i**, n = 4; **ii**, *n* = 6; **iii**, n = 4; **i’**, n = 5); **p < 0.01 *p < 0.05 by Tukey-Kramer LSD

Since Munc18–1-Syntaxin1A interaction was found to be crucial for radial migration during corticogenesis, we examined the role of Syntaxin1A per se in the migration. Syntaxin1A was expressed in the CP, IZ and VZ/SVZ as in the case of Munc18–1 during corticogenesis (Fig. 6a). It was diffusely distributed in the cytoplasm of migrating neurons (Fig. 6b). For the functional analyses, we prepared pSuper-mStx1A(sh-Stx1A), which knocked down exogenous Syntaxin1A but not 1B in COS7 cells (Fig. 6c). Endogenous Syntaxin1A was also knocked, albeit partially, in primary cultured mouse cortical neurons (Fig. 6d). We also made mSyntaxin1A-R resistant to sh-Stx1A-mediated silencing (Fig. 6c). Then, pCAG-RFP was electroporated in utero with the control RNAi vector or sh-Stx1A into the VZ cells of E14.5 mice brains. Although control neurons migrated to the surface of CP at P0, Syntaxin1A-deficient neurons were accumulated in the IZ (Fig. 6e and f). Notably, Syntaxin1A-R rescued the migration defects partially (Fig. 6e and f). Taken together, although the knockdown effects by sh-Stx1A was incomplete, we assume that Syntaxin1A is essential for neuronal migration perhaps downstream of Munc18–1.

### Role of Munc18–1 in Syntaxin1A localization in migrating neurons

**Fig. 7:**
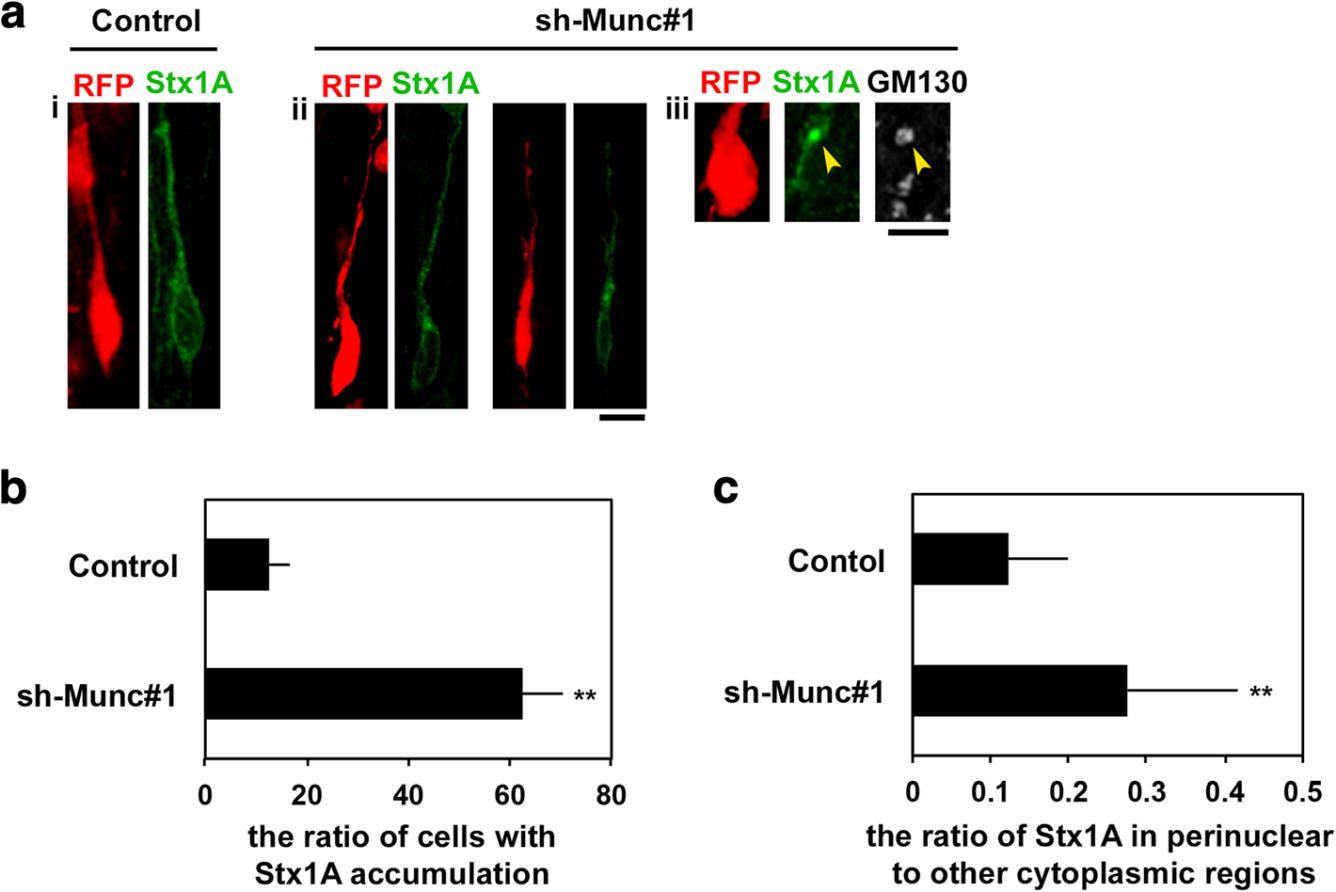
Effects of Munc18–1 knockdown on subcellular distribution of Syntaxin1A**. a** Localization of Syntaxin1A in Munc18–1-deficient cortical neurons. pCAG-RFP was electroporated into E14.5 cerebral cortices with pCAG-GFP-Syntaxin1A **(i)** or with pCAG-GFP-Syntaxin1A + sh-Munc#1 **(ii, iii)**. Coronal sections were prepared at E18.0 and immunostained for GFP-tag **(i, ii)** or GFP-tag plus GM130, a Cis-Golgi marker **(iii)**. Bars in (i-ii), 10 μm and (iii), 5 μm. **b** Quantification of Syntaxin1A accumulation at Golgi. The ratio of cells with GFP-Syntaxin1A accumulation in the lower CP in (**a**). Error bars indicate SD; Control (n = 5), Munc18–1-knockdown (n = 5); **p < 0.01 by Student’s *t*-test. **c** Quantification of subcellular localization of Syntaxin1A in migrating neurons in (**a**). The relative ratio of GFP-Syntaxin1A signal in perinuclear regions with high fluorescent signal to that of other regions (cytoplasm) was evaluated by ImageJ software. The criterion for “perinuclear Syntaxin1A accumulation” is high fluorescence intensity of Syntaxin1A at the perinuclear region, which was defined by ImageJ software. The fluorescent signals of GFP-Syntaxin1A at perinuclear regions and other cytoplasmic regions were calculated with ImageJ software. Error bars indicate SD of five brains containing more than 150 cells. **p < 0.01 by Student’s *t*-test

Munc18–1 has been shown to be critical for plasma membrane localization of Syntaxin1 in neuroendocrine PC12 cells [47]*.* To test if Munc18–1 also regulates subcellular distribution of Syntaxin1A in migrating neurons during corticogenesis, we weakly expressed GFP-Syntaxin1A because it is difficult to distinguish the staining signal of endogenous Syntaxin1A in electroporated neurons from that of surrounding cells. pCAG-RFP was electroporated with pCAG-GFP-Syntaxin1A together with the control vector or sh-Munc#1 into E14.5 mouse brain. When cortical neurons were stained for GFP-tag at E18.0, GFP-Syntaxin1A was distributed throughout the cytoplasm in control migrating neurons, whereas it was aberrantly accumulated around Golgi in Munc18–1-deficient neurons (Fig. 7a and b). The ratio of Syntaxin1A signal in perinuclear regions to that of other cytoplasmic regions was increased in the deficient neurons, compared with the control (Fig. 7c). These results indicated that trafficking of Syntaxin1A from Golgi to the plasma membrane was prevented in Munc18–1-deficient migrating neurons. We assume that Munc18–1 plays an important role in the post-Golgi trafficking of vesicles containing Syntaxin1A, which should be essential for radial migration in the CP.

### Munc18–1 regulates intracellular trafficking of N-Cadherin

**Fig. 8:**
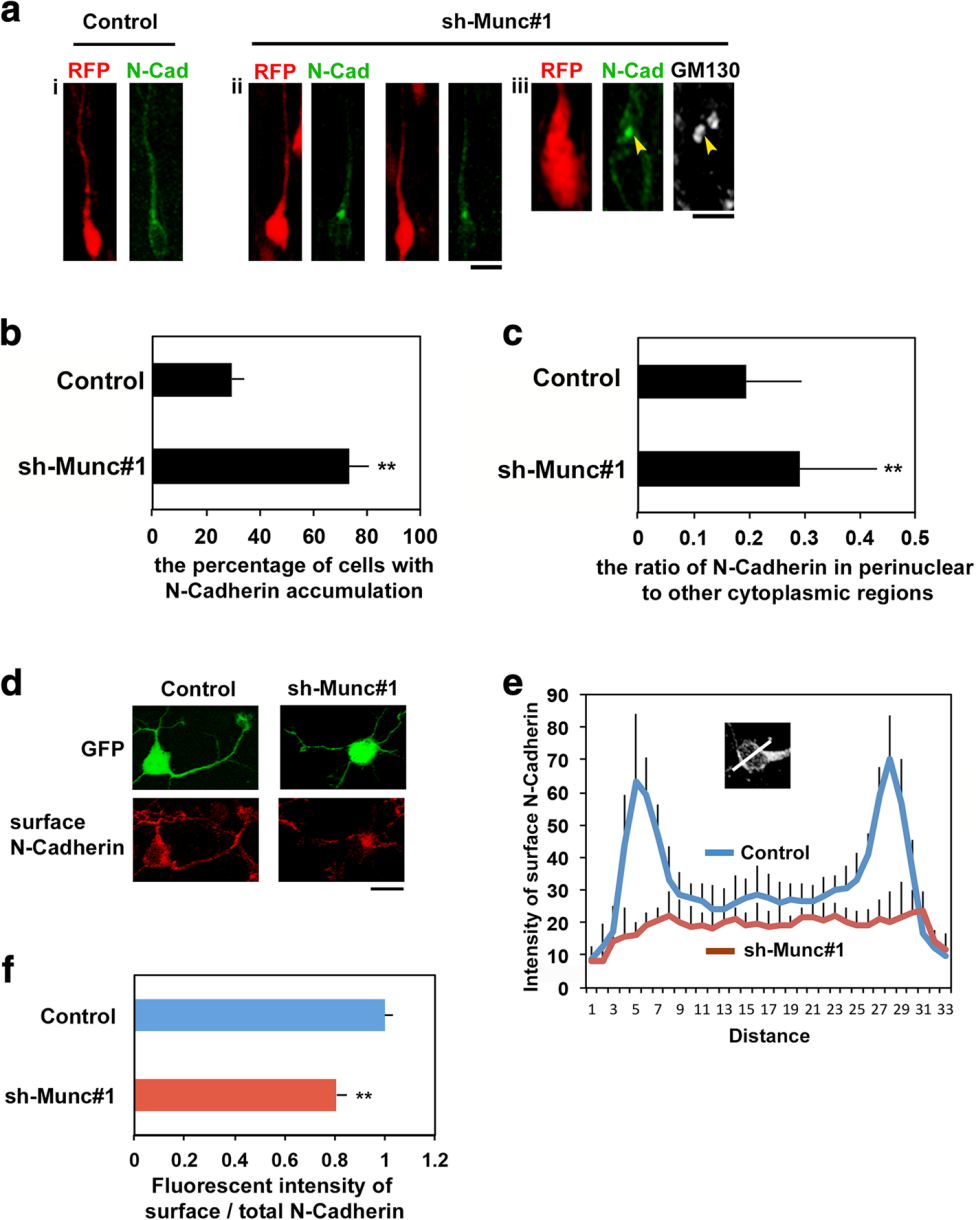
Effects of Munc18–1 knockdown on subcellular distribution of N-Cadherin. **a** Localization of exogenous N-Cadherin in Munc18–1-deficient migrating neurons. E14.5 cerebral cortices were electroporated with pCAG-RFP plus pCAG-HA-N-Cadherin together with pSuper-H1.shLuc **(i)** or sh-Munc#1 **(ii, iii)**. Coronal sections were prepared at E18.0 and immunostained for HA-tag **(i, ii)** or HA-tag plus GM130 **(iii)**. **(c)**. Bars in (**i**-**ii**), 10 μm and (**iii**), 5 μm. **b** Quantification of N-Cadherin accumulation at Golgi. The ratio of RFP-positive cells with the accumulation was calculated for migrating neurons in the lower CP in (**a**). Error bars indicate SD; Control (n = 5), Munc18–1-knockdown (n = 5); **p < 0.01 by Tukey-Kramer LSD. **c** Quantification of subcellular localization of N-Cadherin. The ratio of N-Cadherin in perinuclear to other cytoplasmic regions was analyzed. Error bars indicate SD of 5 brains containing more than 200 cells. **p < 0.01 by Student’s *t*-test. **d** Localization of endogenous N-Cadherin in Munc18–1-deficient cortical neurons. pCAG-GFP was coelectroporated with pSuper-H1.shLuc (Control) or sh-Munc#1 into the E14.5 cerebral cortices. Neurons were isolated at E16.5, cultured for 48 h, fixed and stained with polyclonal anti-N-Cadherin without permeabilization. Bar, 10 μm. **e** Quantification of fluorescence intensity profiles of cell surface N-Cadherin across the cell bodies of control (blue) and Munc18–1-deficient neurons (red). Means +/− SEM (Control, n = 6; sh-Munc#1, n = 7). **f** Quantification of fluorescence intensity profiles of cell surface N-Cadherin in neurites. After the staining as in (**d**), neurons were permeabilized and double-stained with monoclonal anti-N-Cadherin and anti-GFP. Then, the ratio of the fluorescent intensity of surface N-Cadherin to total N-Cadherin was analyzed. Error bars indicate SD in each condition (n = 4). More than 300 neurites were analyzed in each experiment. **p < 0.01 by Student’s *t*-test

Neuronal cadherin (N-Cadherin) is one of the important adhesion molecules for the attachment of migrating neurons to radial glial fibers. Proper localization and cell surface expression of N-Cadherin is required for glial fiber-guided neuronal migration [48, 49]. We thus analyzed the function of Munc18–1 in vesicle transport by focusing on N-Cadherin distribution. Munc18–1-silencing disturbed distribution of HA-N-Cadherin expressed in migrating neurons and caused its abnormal accumulation at Golgi, compared to the diffuse distribution in the cytoplasm in the control cell (Fig. 8a and b). The ratio of HA-N-Cadherin signal in perinuclear regions to that of other cytoplasmic regions also increased in Munc18–1-deficient neurons (Fig. 8c). These phenotypes were very similar to those of Syntaxin1A trafficking in Munc18–1-deficient neurons (Fig. 7), indicating that Munc18–1 may function in the post-Golgi trafficking of Syntaxin1A- and N-Cadherin-containing vesicles.

Since N-Cadherin exerts its function at cell surface, we next analyzed the involvement of Munc18–1 in vesicle fusion at cell surface. To this end, we used primary cultured cortical neurons to detect cell surface N-Cadherin as neurons were too closely packed in cortical slices to dissect. E14 mouse brain was electroporated in utero with pCAG-GFP together with the control RNAi vector or shMunc#1. After isolation at E16, neurons were cultured for 48 h and then stained for cell-surface N-Cadherin without permeabilization, followed by permeabilization for staining total N-Cadherin. Endogenous N-Cadherin was distributed on the surface of control cell bodies, whereas such distribution was suppressed when Munc18–1 was silenced (Fig. 8d and e). Cell surface distribution of N-Cadherin was also decreased in neurites of the deficient neurons as in the case of cell body (Fig. 8f).

Although transport of Syntaxin1A from Golgi to the plasma membrane region was dependent on Munc18–1, Syntaxin1A per se was not required for the post-Golgi vesicle transport when N-Cadherin was used as an indicator (Additional file 9: Figure S5a, b). On the other hand, Syntaxin1A-knockdown suppressed N-Cadherin distribution on the cell surface as in the case of Munc18–1-silencing (Additional file 9: Figure S5c). Syntaxin1A was thus supposed to regulate the vesicle fusion process to ensure proper interaction of migrating neurons with glial fibers during corticogeneis.

Collectively, while Munc18–1 is known to be essential for the vesicle priming/fusion process in the neurotransmitter release, we here clarified that 1) Munc18–1 likely regulates post-Golgi transport of vesicles containing N-Cadherin, a crucial process for radial migration of excitatory neurons during corticogenesis, and 2) subsequent vesicle fusion and distribution of N-Cadherin to migrating cell surface.

## Discussion

It is well accepted that Munc18–1 is a presynaptic chaperone for Syntaxin1 and regulates neurotransmitter release through the modulation of SNARE complex formation in developed neurons [50]. Although Munc18–1-knockout mice showed complete loss of neurotransmitter secretion from synaptic vesicles, corticogenesis was morphologically normal; cortical layer structure, fiber pathways and synapse formation were completed normally [51]. These observations suggest that cortical development including synaptic connectivity does not depend on the Munc18–1 function.

On the other hand, expression of Munc18–1 in developing cerebral cortex suggests its role in cortical development. Critical function of Munc18–1 during brain development also should be approved by *MUNC18–1* gene abnormalities that cause neurodevelopmental disorders such as EIEE, NEE, ID and ASD. Little is, however, known about the significance of MUNC18–1 during brain development and in the above neurodevelopmental disorders.

In the present study, we show that Munc18–1 is involved in excitatory neuron migration during corticogenesis, based on acute knockdown experiments with in utero electroporation. The results obtained may indicate a novel role of Munc18–1 in the embryonic stage where functional synapses were not detected by electron microscopic analyses [51]. The discrepancy between the knockout mice and acute knockdown experiments may be explained by functional redundancy by Munc18 isoforms. Considering that Munc18–2 and Munc18–3 are expressed widely, they may compensate for the loss of Munc18–1 function in the knockout mouse. Notably, Munc18–3 appeared to be essential for brain development since poorly formed axon fibers and mispositioned neurons were observed in the IZ of the null mouse [7]. We assume that acute conditional knockdown of Munc18–1 may circumvent the compensatory effects of general gene-knockout approaches.

We here focused on the pyramidal neurons generated at ~E14.5 which form layer II/III of cerebral cortex. When Munc18–1 was silenced in utero at E14.5 and neuronal migration was monitored by time-lapse imaging from E16.5 for ~24 h, characteristic radial migration delay was observed. Given the absence of functional synapses in E17 mouse neocortex [51], it is plausible that Munc18–1 plays a yet unidentified role in radial migration. The basic molecular mechanism of Munc18–1 function in neuronal migration, however, might be at least partially common to that of the vesicle fusion process in neurotransmitter release of adult neurons, because intracellular vesicle trafficking is required to add new membrane and a variety of molecules to specific regions of migrating cortical neurons [40, 52]. Our results suggest that, when Syntaxin1A or N-Cadherin was used as a tracer, Munc18–1 regulates post-Golgi vesicle trafficking to the plasma membrane and subsequent vesicle fusion at cell surface in migrating neurons during corticogenesis.

Recent study reported that an epilepsy-causative C180Y mutation in *MUNC18–1* destabilizes protein structure and induces protein degradation through the proteasome in vitro [18]. Since Munc18–1 mutants analyzed here were severely degradated in cells, these mutations appeared to have loss-of-function effects. On the other hand, since it has been reported that Munc18–1 controls aggregative propensity of α-synuclein and that C180Y mutation induces abnormal aggregation of α-synuclein, the migration defects observed in this study might be attributable to the level of toxic α-synuclein aggregation [17]. In this context, disrupted function of Munc18–1 has been shown to trigger neuronal degeneration [53, 54]. Given that clinical symptoms are different in respective patients with *MUNC18–1* gene abnormalities, additional environmental or genetic factors affecting neurodegeneration are suggested in each patient.

Previous works using knockout mice and dominant-negative mutants revealed that Cdk5 plays an essential role in cortical neuron migration through regulation of cell morphology and polarity [55, 56]. On the other hand, experiments using chemical inhibitors suggest a role of PKC in the migration, although underlying molecular mechanism has been obscure [46]. The results obtained here suggest that phsophorylation by PKC is required for Munc18–1 function in cortical neuron migration. Since the Munc18–1 mutations at the PKC-phosphorylation sites that mimic phosphorylated or unphosphorylated state both demonstrated similar effects on cortical neuron migration, balance between the phosphorylation and unphosphorylation states and/or dynamic changes of the phosphorylation status might be essential for the regulation of Munc18–1 function during the migration. Given that PKC-mediated phosphorylation is crucial for Munc18–1 localization in nerve terminals in developed neurons [43], PKC may be involved in the localization of Munc18–1 at specific intracellular sites, where Munc18–1 exerts its functions for proper radial migration.

Accumulating evidence supports the hypothesis that Syntaxin1A and B play important roles in brain development. As for Syntaxin1B, mutations such as truncation, in-frame insertion and deletion have been reported to cause fever-associated epilepsy syndromes with a wide phenotypic spectrum, ranging from simple febrile seizures to severe epileptic encephalopathies [57]. Since these syndromes usually emerge from age of ~6-months, functional defects in the neurotransmitter release are possible to be the major cause of the syndromes. Meanwhile, Syntaxin1A is also related to neuronal development since its single nucleotide polymorphisms (SNPs) are significantly associated with ASD [58, 59]. Based on these observations, both Syntaxin1A and B seemingly take part in cortical development. Considering different implication of Syntaxin1A and B in neurodevelopmental disorders, they might have temporally different roles during brain development and impaired Syntaxin1A function might be related to the clinical features common to those of *MUNC18–1* gene abnormalities. In this study, only Syntaxin1A showed rescue effects on Munc18–1-silencing, implying specific functional relationship of Syntaxin1A with Munc18–1 during radial migration. However, it should be noted that other mechanism(s) could contribute to the neurodevelopmental defects observed in this study, since the rescue effect on Munc18–1-silencing by Syntaxin1A expression were inefficient compared to that of Munc18–1R expression.

**Fig. 9:**
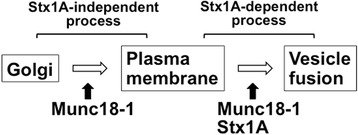
Current working hypothesis of Munc18–1 function during corticogenesis

In the present study, we clarified that Munc18–1 plays an essential role in neuronal migration during corticogenesis. During radial migration, Munc18–1 is considered to regulate post-Golgi vesicle trafficking in a Syntaxin1A-independent manner. Munc18–1 is then likely to control the vesicle fusion step in harmony with Syntaxin1A to ensure distribution of numerous proteins including N-Cadherin, which are essential for radial migration (Fig. 9). Although PKC-mediated phosphorylation is seemingly crucial for cortical neuron migration, its molecular mechanism is still enigmatic. Further investigation is required to clarify the MUNC18–1 function in corticogenesis and pathogenesis of neurodevelopmental disorders with *MUNC18–1* gene abnormalities.

## Conclusions

During corticogenesis, Munc18-1 (Stxbp1) regulates radial migration by modulating vesicle transport from Golgi to the plasma membrane and subsequent fusion, in order to distribute various proteins on the cell surface. Syntaxin1A may participate in the fusion step under the control of Munc18-1. Gene abnormalities in *MUNC18-1* may result in abnormal corticogenesis, leading to neonatal epileptic encephalopathy and other neurodevelopmental disorders.

## Additional files

Additional file 1: Figure S1. Quality check of anti-Munc18–1 antibody. Coronal sections (E17) were stained with (*upper* panels) or without (*lower* panels) anti-Munc18–1. Nuclei (blue) were visualized with DAPI. Bar, 100 μm. (TIFF 28233 kb)

Additional file 2: Figure S2. Role of Munc18–1 in the terminal translocation of migrating neurons. Cerebral cortices were electroporated with pCAG-RFP together with pSuper-H1.shLuc (Control) or sh-Munc#1 at E15.5. Coronal sections were prepared at P3, and stained for RFP (white) and nuclei (blue). Dotted lines represent the pial surface (*upper*) and the top of CP (*lower*). MZ, marginal zone; PCZ, primitive cortical zone. Bar, 10 μm. (TIFF 27091 kb)

Additional file 3: Video 1. Time-lapse imaging of control cortical neurons migrating in upper IZ - lower CP. (ZIP 12000 kb)

Additional file 4: Video 2. Time-lapse imaging of Munc18–1-defcient cortical neurons stranded in upper IZ - lower CP. (ZIP 15000 kb)

Additional file 5: Video 3. Time-lapse imaging of control cortical neurons migrating in CP. (ZIP 25000 kb)

Additional file 6: Video 4. Time-lapse imaging of Munc18–1-defcient cortical neurons migrating in CP. (ZIP 26000 kb)

Additional file 7: Figure S3. Effects of Munc18–1-silencing on cell division in the VZ. (a) Effects of Munc18–1-silencing on EdU incorporation. E14.5 cortices were coelectroporated with pCAG-GFP together with pSuper-H1.shLuc (Control) or sh-Munc#1. Coronal sections were visualized for GFP (green) and EdU (red). Arrowheads indicate EdU/GFP double-positive cells. Dotted lines represent the ventricular surface. Bar, 10 μm. (b) Quantification of EdU/GFP double-positive cells among GFP-positive ones in (a). Error bars indicate SD, and *n* = 4. (TIFF 27842 kb)

Additional file 8: Figure S4. Effects of Cdk5- and PKC-mediated phosphorylation of Munc18–1 on neuronal migration during corticogenesis. (a, b) Effects of phosphorylation of Munc18–1 by Cdk5 (a) or by PKC (b). pCAG-RFP was electroporated with pSuper-H1.shLuc (Control) or sh-Munc#1 together with pCAG vector (#1 + vector), pCAG-Myc-mMunc18–1R (#1 + WT), −mMunc18–1-Thr574Ala (#1 + T574A), −mMunc18–1-Thr574Asp (#1 + T574D), −mMunc18–1-Ser306Ala (#1 + S306A), Ser313Ala (#1 + S313A) or -mMunc18–1-Ser306Asp (#1 + S306D), Ser313Asp (#1 + S313D) into cerebral cortices at E14.5, followed by fixation at P2. Quantification of the distribution of neurons in distinct regions of the cerebral cortex for each condition was analyzed as in Fig. 2d. Error bars indicate SD (Control, *n* = 5; #1 + vector, n = 4; #1 + WT, *n* = 7; #1 + T574A, n = 4; #1 + T574D, n = 5; #1 + S306A, n = 7; #1 + S313A, n = 7; #1 + S306D, *n* = 6; #1 + S313D, n = 7); * *p* < 0.05, ** *p* < 0.01 by Tukey-Kramer LSD. (c) Expression profiles of #1 + T574A, #1 + T574D, #1 + S306A, #1 + S306D, #1 + S313A and #1 + S313D in (a) and (b). RFP (red) and Myc-tag (green) were stained. Bar, 5 μm. (TIFF 28944 kb)

Additional file 9: Figure S5. Localization of N-Cadherin in Syntaxin1A-deficient migrating neurons. (a) E14.5 cerebral cortices were electroporated with pCAG-RFP plus pCAG-HA-N-Cadherin together with pSuper-H1.shLuc (i) or sh-Stx1A (ii). Coronal sections were prepared at E18.0 and immunostained for HA-tag. Bar, 5 μm. (b) Quantification of N-Cadherin accumulation at Golgi. The ratio of RFP-positive cells with the accumulation was calculated for migrating neurons in the lower CP in (a). Error bars indicate SD. (Control, n = 6; sh-Stx1A, n = 6) (c) Quantification of fluorescence intensity profiles of cell surface N-Cadherin across the cell bodies of control (blue) and the deficient neurons (red). Means +/− SEM (Control, 42 neurons; sh-Stx1A, 70 neurons). (TIFF 26845 kb)
