# Supplementary figures and images for: MUNC18–1 gene abnormalities are involved in neurodevelopmental disorders through defective cortical architecture during brain development
Source: Acta Neuropathol Commun. 2017 Nov 30;5:92. doi: 10.1186/s40478-017-0498-5 (PMC5709915; doi:10.1186/s40478-017-0498-5)

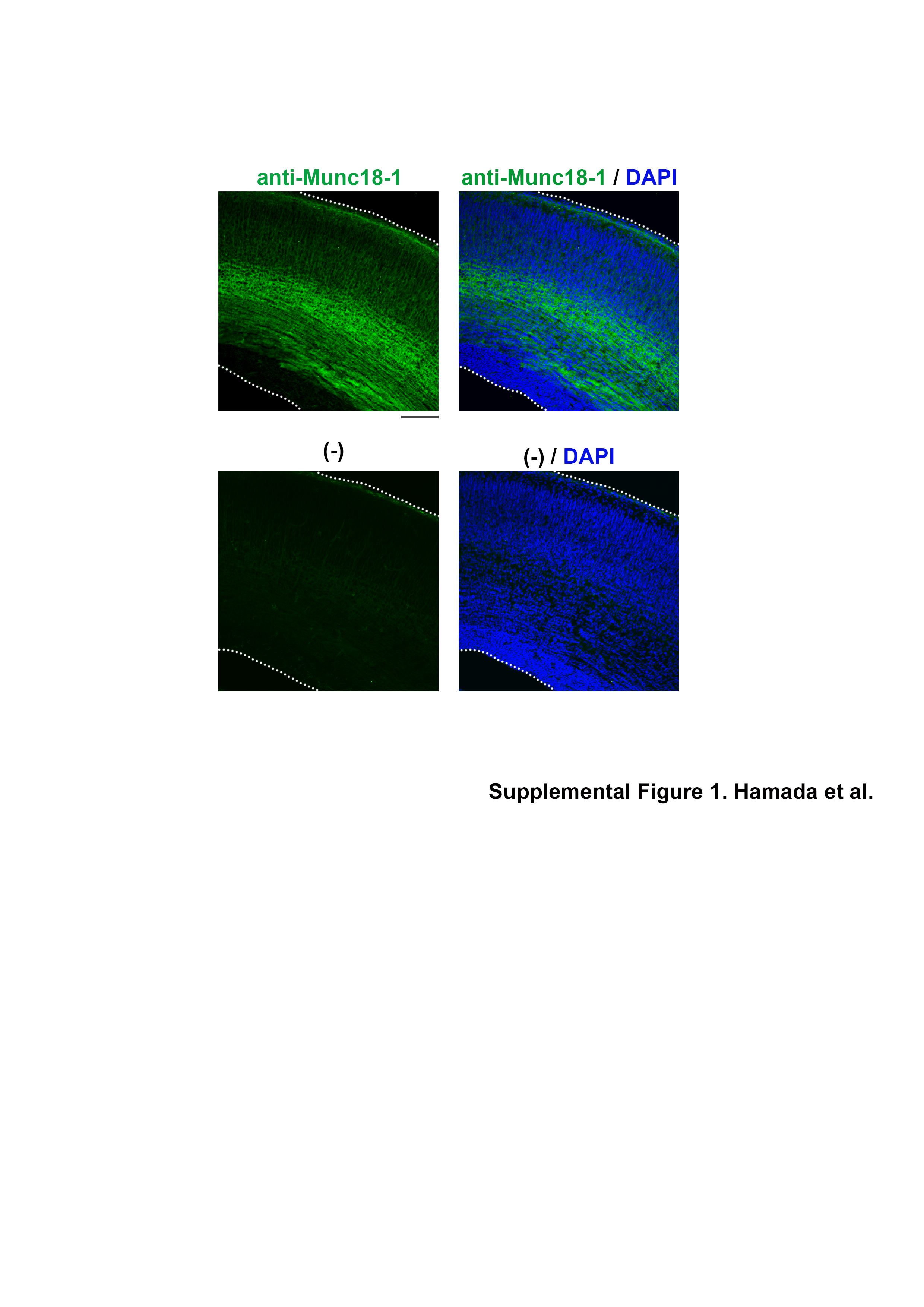

Supplement: Supplementary file 1 — Quality check of anti-Munc18–1 antibody. Coronal sections (E17) were stained with (upper panels) or without (lower panels) anti-Munc18–1. Nuclei (blue) were visualized with DAPI. Bar, 100 μm. (TIFF 28233 kb) [file 40478_2017_498_MOESM1_ESM.tif]

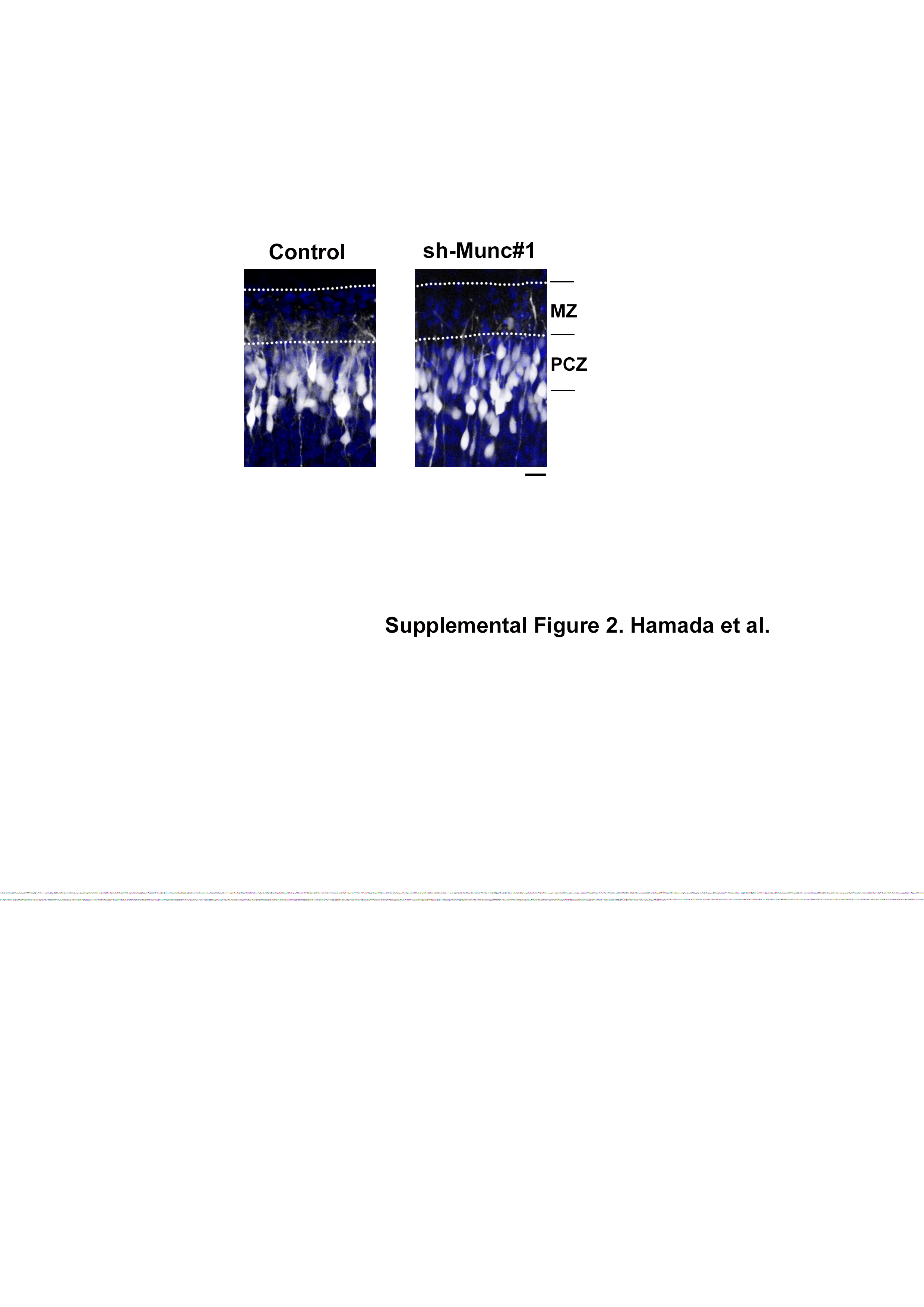

Supplement: Supplementary file 2 — Role of Munc18–1 in the terminal translocation of migrating neurons. Cerebral cortices were electroporated with pCAG-RFP together with pSuper-H1.shLuc (Control) or sh-Munc#1 at E15.5. Coronal sections were prepared at P3, and stained for RFP (white) and nuclei (blue). Dotted lines represent the pial surface (upper) and the top of CP (lower). MZ, marginal zone; PCZ, primitive cortical zone. Bar, 10 μm. (TIFF 27091 kb) [file 40478_2017_498_MOESM2_ESM.tif]

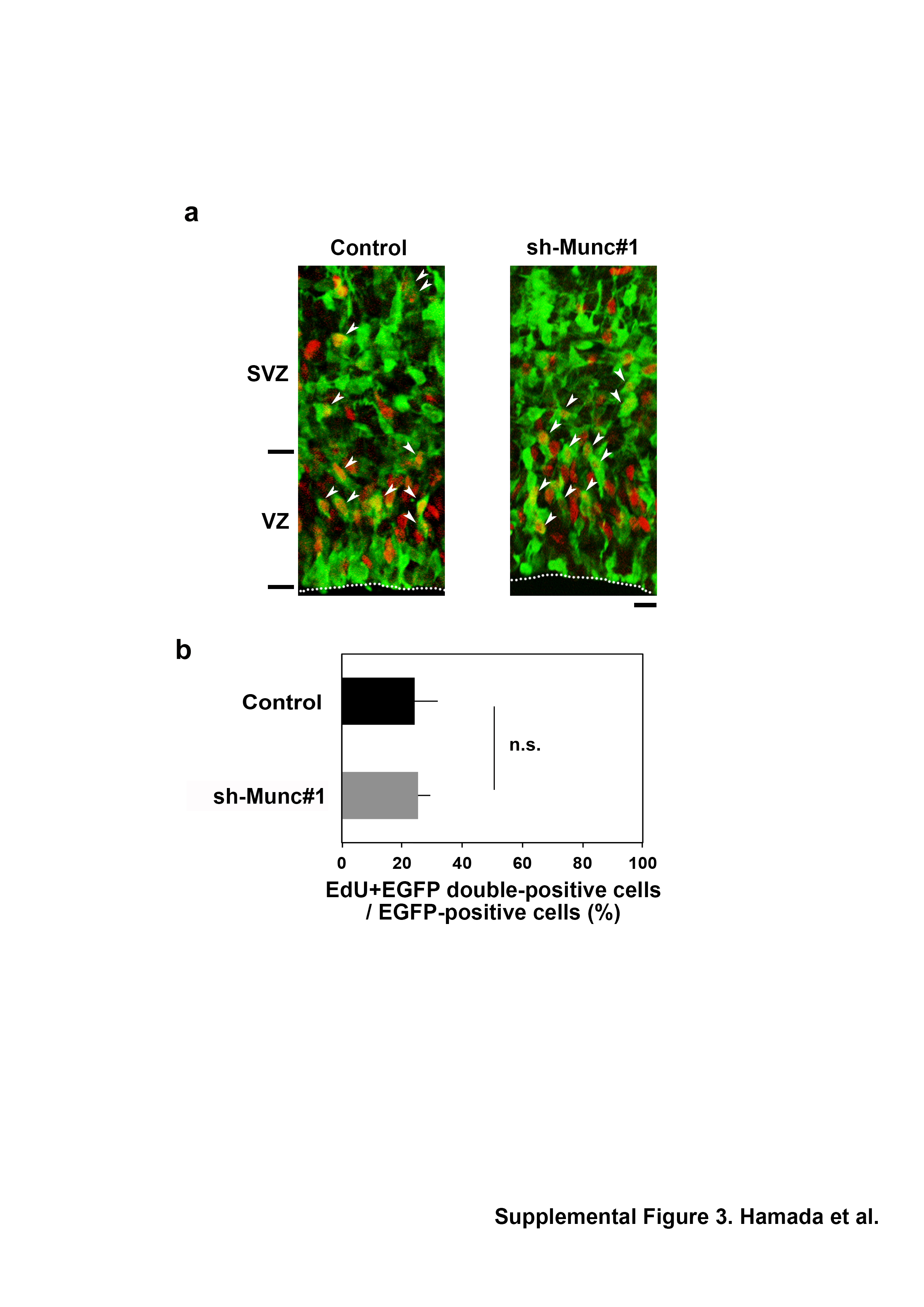

Supplement: Supplementary file 7 — Effects of Munc18–1-silencing on cell division in the VZ. (a) Effects of Munc18–1-silencing on EdU incorporation. E14.5 cortices were coelectroporated with pCAG-GFP together with pSuper-H1.shLuc (Control) or sh-Munc#1. Coronal sections were visualized for GFP (green) and EdU (red). Arrowheads indicate EdU/GFP double-positive cells. Dotted lines represent the ventricular surface. Bar, 10 μm. (b) Quantification of EdU/GFP double-positive cells among GFP-positive ones in (a). Error bars indicate SD, and n = 4. (TIFF 27842 kb) [file 40478_2017_498_MOESM7_ESM.tif]

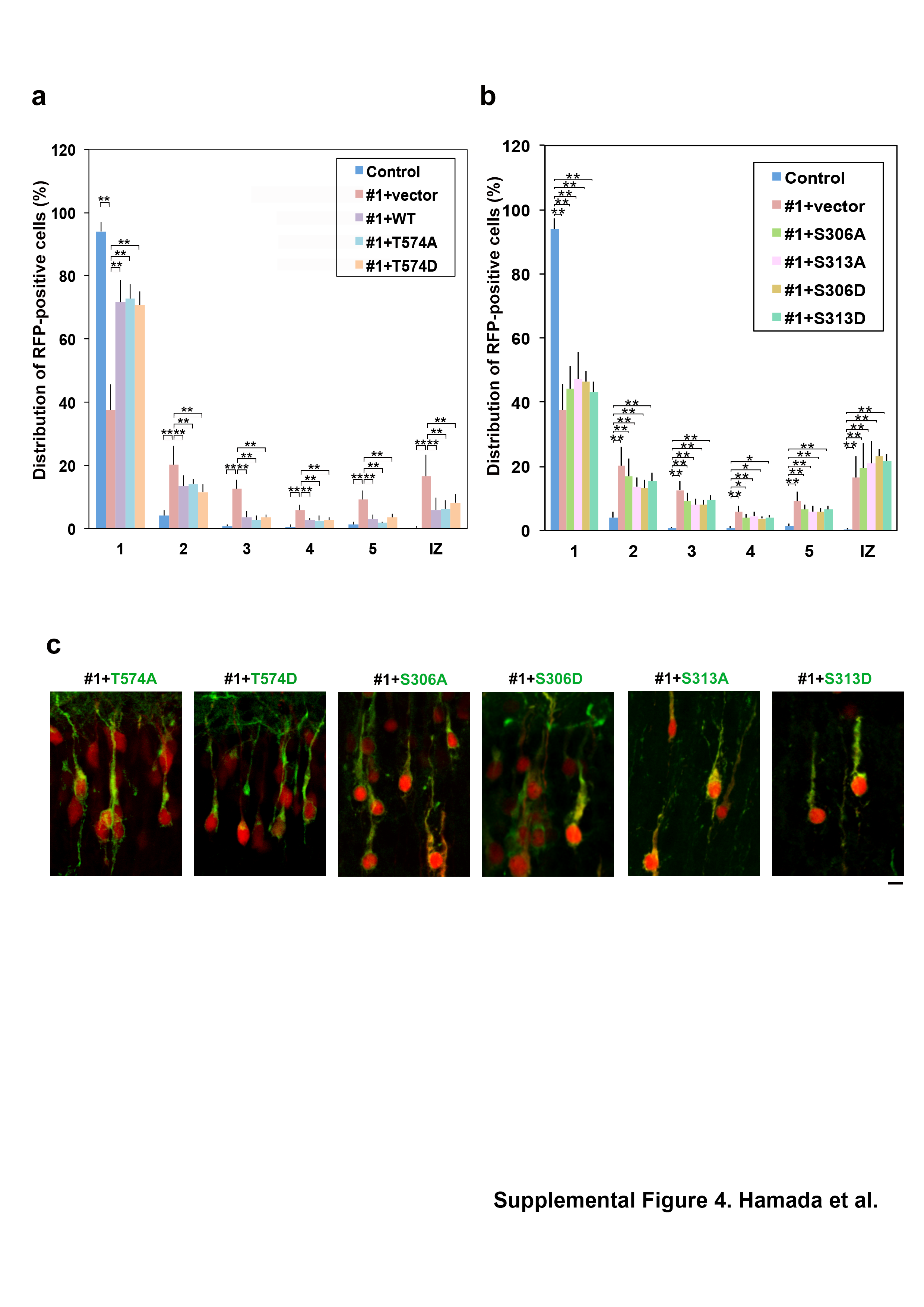

Supplement: Supplementary file 8 — Effects of Cdk5- and PKC-mediated phosphorylation of Munc18–1 on neuronal migration during corticogenesis. (a, b) Effects of phosphorylation of Munc18–1 by Cdk5 (a) or by PKC (b). pCAG-RFP was electroporated with pSuper-H1.shLuc (Control) or sh-Munc#1 together with pCAG vector (#1 + vector), pCAG-Myc-mMunc18–1R (#1 + WT), −mMunc18–1-Thr574Ala (#1 + T574A), −mMunc18–1-Thr574Asp (#1 + T574D), −mMunc18–1-Ser306Ala (#1 + S306A), Ser313Ala (#1 + S313A) or -mMunc18–1-Ser306Asp (#1 + S306D), Ser313Asp (#1 + S313D) into cerebral cortices at E14.5, followed by fixation at P2. Quantification of the distribution of neurons in distinct regions of the cerebral cortex for each condition was analyzed as in Fig. 2d. Error bars indicate SD (Control, n = 5; #1 + vector, n = 4; #1 + WT, n = 7; #1 + T574A, n = 4; #1 + T574D, n = 5; #1 + S306A, n = 7; #1 + S313A, n = 7; #1 + S306D, n = 6; #1 + S313D, n = 7); * p < 0.05, ** p < 0.01 by Tukey-Kramer LSD. (c) Expression profiles of #1 + T574A, #1 + T574D, #1 + S306A, #1 + S306D, #1 + S313A and #1 + S313D in (a) and (b). RFP (red) and Myc-tag (green) were stained. Bar, 5 μm. (TIFF 28944 kb) [file 40478_2017_498_MOESM8_ESM.tif]

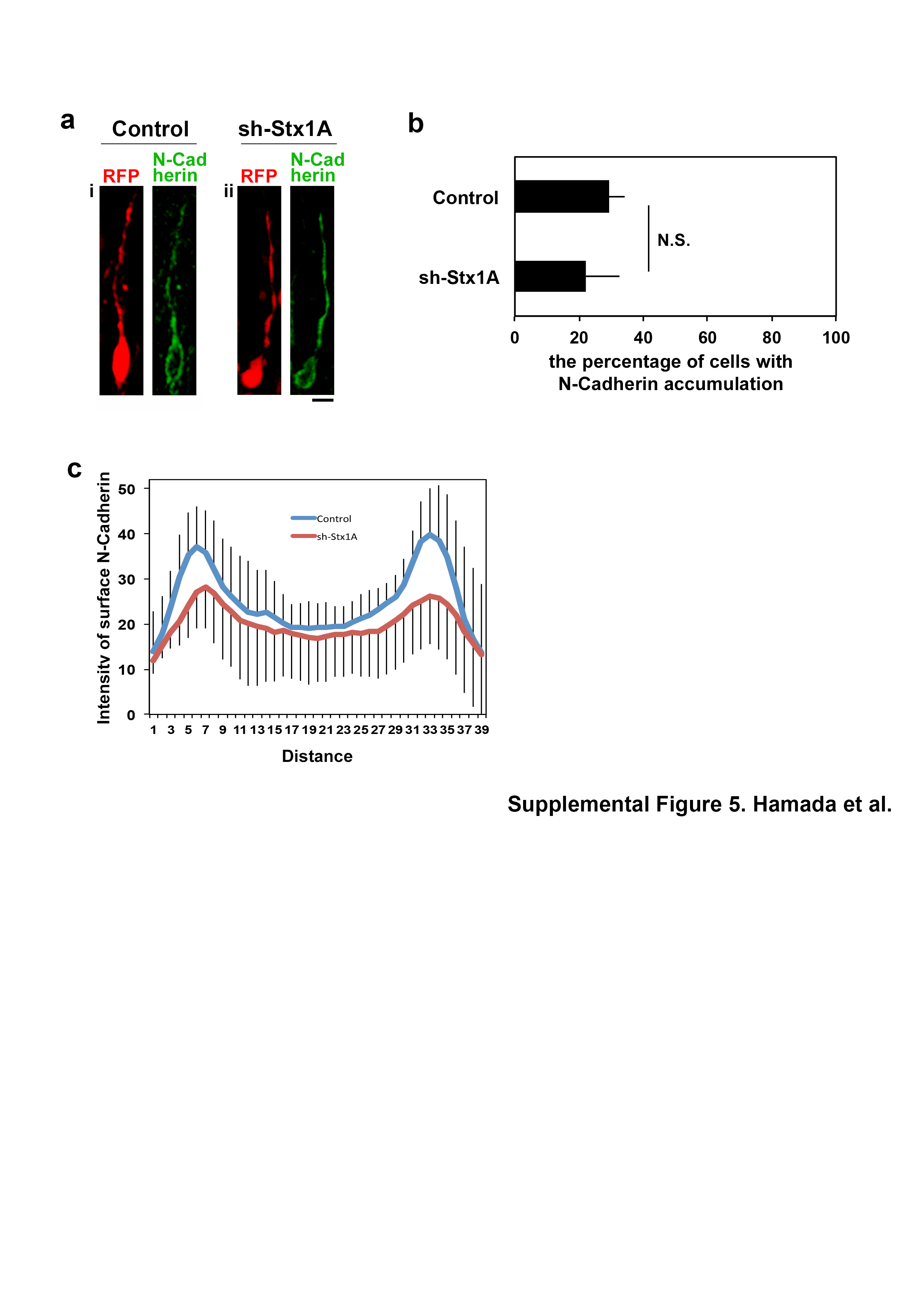

Supplement: Supplementary file 9 — Localization of N-Cadherin in Syntaxin1A-deficient migrating neurons. (a) E14.5 cerebral cortices were electroporated with pCAG-RFP plus pCAG-HA-N-Cadherin together with pSuper-H1.shLuc (i) or sh-Stx1A (ii). Coronal sections were prepared at E18.0 and immunostained for HA-tag. Bar, 5 μm. (b) Quantification of N-Cadherin accumulation at Golgi. The ratio of RFP-positive cells with the accumulation was calculated for migrating neurons in the lower CP in (a). Error bars indicate SD. (Control, n = 6; sh-Stx1A, n = 6) (c) Quantification of fluorescence intensity profiles of cell surface N-Cadherin across the cell bodies of control (blue) and the deficient neurons (red). Means +/− SEM (Control, 42 neurons; sh-Stx1A, 70 neurons). (TIFF 26845 kb) [file 40478_2017_498_MOESM9_ESM.tif]
